# Preparation and Performance Analysis of Ag/ZnO Humidity Sensor

**DOI:** 10.3390/s21030857

**Published:** 2021-01-28

**Authors:** Peng Li, Shuguo Yu, Hongyan Zhang

**Affiliations:** School of Physical Science and Technology, Xinjiang University, Urumqi 830046, China; lip@xju.edu.cn (P.L.); yushuguo0818@163.com (S.Y.)

**Keywords:** Ag-modified ZnO, humidity sensor, response, light

## Abstract

Highly sensitive silver (Ag) modified zinc oxide (ZnO) humidity sensors were prepared by hydrothermal synthesis and the mechanism was studied. Experimental results show that Ag-modified ZnO can effectively enhance the performance of a humidity sensor. Large number of oxygen vacancies and many active sites are generated on the surface when molar ratio of Ag^+^ to Zn^2+^ is 1:100, which can accelerate the decomposition of water molecules on surface of the material, thereby improving the response of humidity sensor. Moreover, the linearity of ZnO humidity sensor is greatly improved by silver nanoparticles. Compared with previously reported ZnO-based humidity sensors, Ag/ZnO humidity sensors have a better response (151,700%), good linearity, low hysteresis (3%), and short response/recovery time (36/6 s). At the same time, it is found that the light had little effect on the performance of Ag/ZnO. Therefore, this kind of ZnO sensor with stable performance and excellent performance is expected to be used in the detection of relative humidity in conventional environments.

## 1. Introduction

Recently, humidity sensors have been developed rapidly and have attracted much attention in many fields such as agriculture, food safety, industrial production, medical treatment and so on [1,2,3]. Among the many sensor types, resistive sensors are the most common sensitive components. The applicability is greatly expanded due to its simple manufacturing, high detection sensitivity, and low production cost. A resistive humidity sensor is obtained by covering a substrate with a film made of a humidity-sensitive material. The resistance of the component will change when a water molecule is adsorbed onto the sensitive film under different relative humidity environments, so as to achieve the purpose of relative humidity detection [4,5]. In terms of the structure of the resistance humidity sensor, the selection of sensitive materials is still the key to improving the performance of the relative humidity sensor. Recently, metal oxides, polymers, and carbon materials are often used in the design and manufacture of humidity sensors. Among many materials, SnO_2_ [6,7], TiO_2_ [8,9], WO_3_ [10,11], ZnO [12,13] and other metal oxide semiconductor materials [14,15,16] are widely used in the preparation of sensors because of their simple preparation process, low price, wide source of raw materials and good biological compatibility. Among them, ZnO nanostructures have become a potential sensing material due to their high activity and large specific surface area [17,18], which has attracted widespread attention. Furthermore, ZnO nanostructures exhibit morphologically dependent sensing characteristics [19,20], so the morphological structure also plays a key role in the performance of ZnO sensors.

ZnO as a sensitive material shows poor linearity and low sensitivity for relative humidity detection, which limits its application as a humidity sensor. With the in-depth research requiring the sensitivity of the humidity sensor, the linearity stability needs to be further improved. To improve the responsiveness of ZnO humidity sensors, noble metal doping (Au, Ag, Pd and Pt) is a frequently used method [21,22]. Silver (Ag) is one of the most conductive materials, and its low cost and good catalytic performance have been widely used in sensors. The introduction of modified silver nanoparticles into ZnO can control the surface morphology and crystal structure of ZnO, which is expected to improve the performance of humidity sensors. Moreover, after Ag is introduced into ZnO, the adsorption sites on the surface of the material will increase, and the number of surface defects will increase. On the one hand, large adsorption sites and high surface defects can make water molecules adsorbed on surface of the material decompose quickly, and hence improves the response speed of the sensor. On the other hand, silver particles have good electrical conductivity, which is beneficial to improve linearity of the sensor, so Ag/ZnO composite material can provide a new idea for the preparation of high-performance humidity sensors.

In this work, we successfully prepared an Ag/ZnO humidity sensor using the hydrothermal method, and response of the sensor under different light conditions was studied. Experimental results show that an Ag/ZnO humidity sensor exhibits high sensitivity, low hysteresis and short response/recovery time when a molar ratio of Ag^+^ to Zn^2+^ is 1:100. This is mainly because in this case there are a large number of oxygen vacancies and active sites on the surface of ZnO. Under the action of oxygen vacancies and active sites, the water molecules on the surface of the Ag/ZnO-2 humidity sensor are rapidly decomposed, thereby increasing response of the humidity sensor. Compared with pure ZnO, such a relative humidity sensor based on modified silver has better linearity.

## 2. Experimental

### 2.1. Experimental Materials and Test Equipment

The main materials and reagents used in this experiment were zinc acetate dihydrate (Zn (CH_3_COOH)_2_·2H_2_O), ethanol (C_2_H_5_OH), ethanolamine (MEA) and silver nitrate hexahydrate (AgNO_3_·6H_2_O). All the above materials were purchased from Sangon Biotech (Shanghai, China, www.sangon.com). The chemical reagents used were of analytical grade, and deionized water (DI) was used throughout the experiments. Morphology of the sample was tested by a field emission scanning electron microscope (FESEM (Hitachi, Japan)). Crystal structure of the material was tested by an X-ray powder diffractometer XRD (Bruker, Karlsruhe, Germany). Absorption spectrum was tested by UV-Vis (PerkinElmer, Waltham, MA, USA) spectrophotometer. Elemental composition of the sample was analyzed by X-ray photoelectron spectroscopy (XPS) (Thermo Fisher Scientific Corporation, Waltham, MA, USA). The electrochemical characteristics of the humidity sensor were tested on the Zennium workstation (CIMPS-2, Zahner, Kronach, Germany).

### 2.2. Preparation of ZnO Microparticles

All reagents were of analytical grade without further purification. The specific experimental details are described as follows. Zinc acetate dihydrate (0.230 g) and sodium hydroxide (0.364 g) were mixed together in 25 mL of deionized water and 10 mL of ethanol was magnetically stirred at 65 °C for 10 min to form a transparent solution. 1 mL of MEA was added dropwise to the above mixed solution and stirred for 2 h. Finally, the gel was dried in a 60 °C drying oven for 2 h, and the annealing process was carried out in a tube furnace at 600 °C for 2 h under nitrogen protection.

### 2.3. Preparation of Ag-doped ZnO Microparticles (Ag/ZnO)

In total, 2.3 g of Zn (CH_3_COOH)_2_·2H_2_O and AgNO_3_ with different molar ratios (molar ratio of Ag+ to Zn^2+^ is 1:10, 1:100, 1:1000) were dissolved into a mixed solution of 25 mL of deionized water and 10 mL of ethanol, heated up to 65 °C with magnetically stirring for 10 min, 1 mL of ethanolamine (MEA) was added and magnetically stirred for 2 h to obtain a uniform white sol, which was left to stand at room temperature for 48 h. Then, the gel was dried in a 60 °C drying oven for 2 h. Finally, chemical vapor deposition (CVD) was used to anneal for 2 h at 600 °C under nitrogen protection in a tube furnace. We named the above samples Ag/ZnO-1, Ag/ZnO-2 and Ag/ZnO-3, respectively.

### 2.4. Relative Humidity Sensitive Characteristics Test

**Figure 1:**
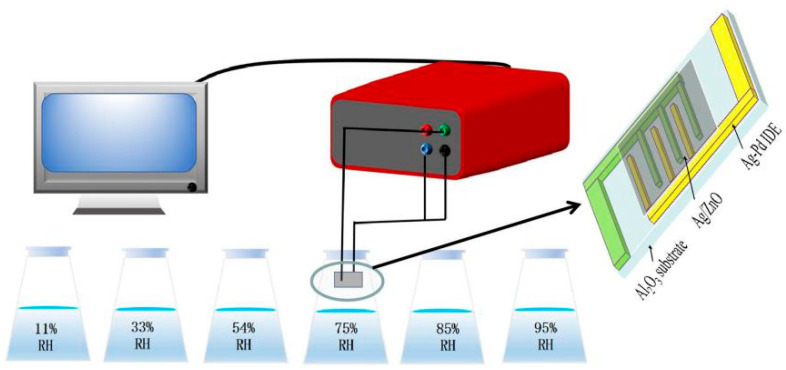
The testing process of the Ag/ZnO humidity sensor.

During the measurement of humidity characteristics, different humidity environments were controlled by saturated salt solutions of LiCl, MgCl_2_, Mg(NO_3_)_2_, NaCl, KCl and KNO_3_, corresponding to relative humidity of 11%, 33%, 54%, 75%, 85% and 95% [19]. In conventional humidity measurement, humidity usually refers to relative humidity, which is a generally accepted method of measuring humidity, and so in this article, relative humidity is used for measurement. Sprayed ZnO, Ag/ZnO and water was mixed on the Ag-Pd interdigital electrode (IDE) and then put it in a 60 °C constant temperature oven to dry for 1 h to form a humidity sensor. Throughout the measurement processes, the test voltage was set to be AC 1V, measurement frequency was 40 Hz to 100 kHz, and the whole test process was carried out at room temperature (25 °C). The test details of the humidity sensor are shown in Figure 1.

## 3. Results and Discussion

**Figure 2:**
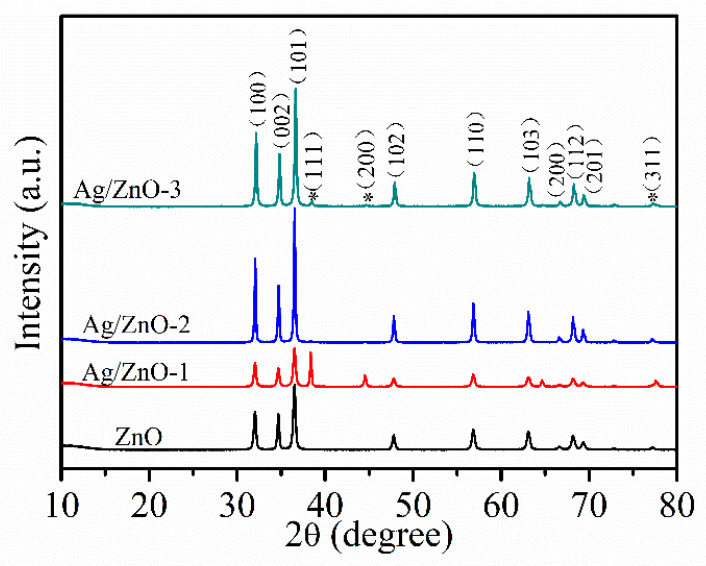
XRD spectra of ZnO, Ag/ZnO-1, Ag/ZnO-2 and Ag/ZnO-3.

Figure 2 shows XRD patterns of ZnO, Ag/ZnO-1, Ag/ZnO-2 and Ag/ZnO-3, which can be used to analyze crystal structures of all the samples. The diffraction peaks of all samples at 2θ of 31.8°, 34.2°, 36.3°, 47.5°, 56.7°, 62.7°, 66.4°, 68.1° and 69.1° are similar to the ones of typical hexagonal wurtzite structures consistent (JCPDS No. 36–1451). Compared with ZnO, the weaker diffraction peaks corresponding to (111), (200), and (311) crystal planes in Ag/ZnO-1, Ag/ZnO-2 and Ag/ZnO-3 belong to Ag crystals of face centered cubic (fcc) structure. The appearance of (111), (200), and (311) crystal plane diffraction peaks indicate the successful recombination of Ag and ZnO. Compared with Ag/ZnO-1, the intensity of diffraction peak of Ag in Ag/ZnO-2 and Ag/ZnO-3 gradually becomes weaker, which is mainly due to the gradually decreasing Ag content in the sample.

**Figure 3:**
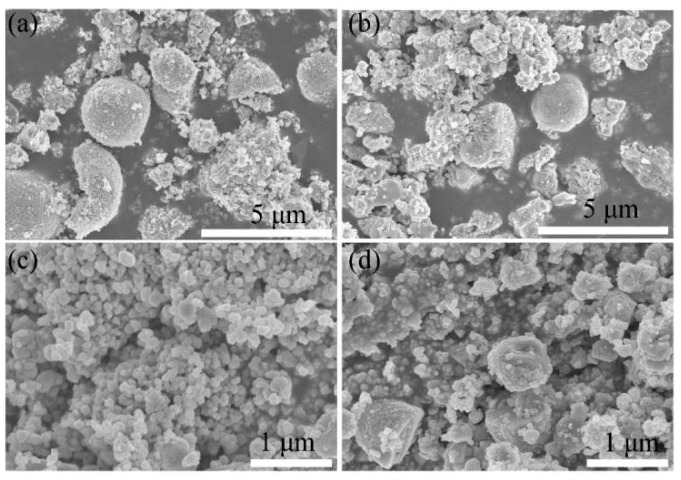
SEM of (**a**) ZnO and (**b**) Ag/ZnO-1, (**c**) Ag/ZnO-2 and (**d**) Ag/ZnO-3.

Figure 3 shows the scanning electron microscope images of ZnO and Ag/ZnO. In Figure 3a,b, ZnO and Ag/ZnO-1 exhibit irregular microparticles with a diameter of about 50–70 nm and 130–160 nm, and these irregular microparticles are agglomerated. Ag/ZnO-2 are irregular microparticles with a diameter of about 70–90 nm, and the particles are evenly distributed and dispersed without agglomeration (Figure 3c). The structure of Ag/ZnO-2 is evenly distributed and more dispersed. Compared with the other three structures, it can provide more adsorption sites for water molecules, making more water molecules adsorb to the surface of the material. This structure plays a vital role in the humidity sensor. In Figure 3d, Ag/ZnO-3 is also of irregular particles with a diameter of about 60–70 nm, and the particles are slightly agglomerated. In general, compared with the other three structures, Ag/ZnO-2 may show better humidity sensing performance.

**Figure 4:**
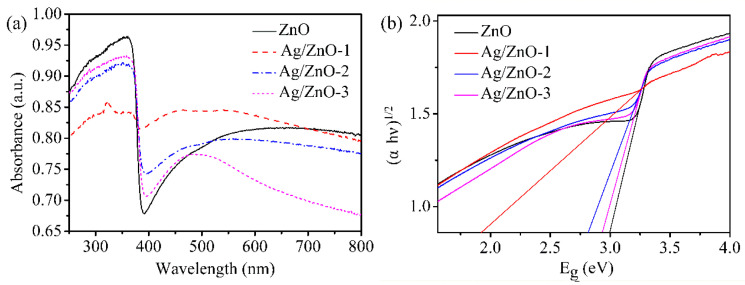
(**a**) UV-vis absorption spectrum and (**b**) E_g_ of ZnO, Ag/ZnO-1, Ag/ZnO-2 and Ag/ZnO-3.

In Figure 4, the optical absorption characteristics of all the samples are given by UV-vis absorption spectrum. In the ultraviolet region as shown in Figure 4a, the strong absorptions for all of ZnO, Ag/ZnO-1, Ag/ZnO-2 and Ag/ZnO-3 indicate that all the samples are wide bandgap direct semiconductors. The strong absorption peak located at 350 nm belongs to intrinsic absorption of ZnO. Because ZnO has strong absorption in ultraviolet light and weak absorption in visible light, there is a sharp decrease at 390 nm. Compared with ZnO, there is blue shift for all the absorption peaks of Ag/ZnO-1, Ag/ZnO-2 and Ag/ZnO-3, which indicates that the band gap of the samples gradually becomes smaller. The absorption peaks of Ag/ZnO-1, Ag/ZnO-2 and Ag/ZnO-3 at 480 nm are gradually weakened with the decreasing of Ag content. Figure 4b shows the band gaps of ZnO, Ag/ZnO-1, Ag/ZnO-2 and Ag/ZnO-3 calculated by the Kubelka–Munk formula (αhν)^2^ = A (hν − E_g_), where α is the absorption coefficient, hν is the photon energy, E_g_ is the band gap energy, and A is a constant. The band gaps of ZnO, Ag/ZnO-1, Ag/ZnO-2 and Ag/ZnO-3 are 3.00 eV, 1.94 eV, 2.80 eV and 2.92 eV, respectively. With the increase of the doped Ag content, the forbidden band width of the sample gradually decreases, which is consistent with the analysis result as in Figure 4a. The lower the band gap energy of the material, the lower the energy it should generate, which is beneficial to improving the conductivity of the humidity sensor.

**Figure 5:**
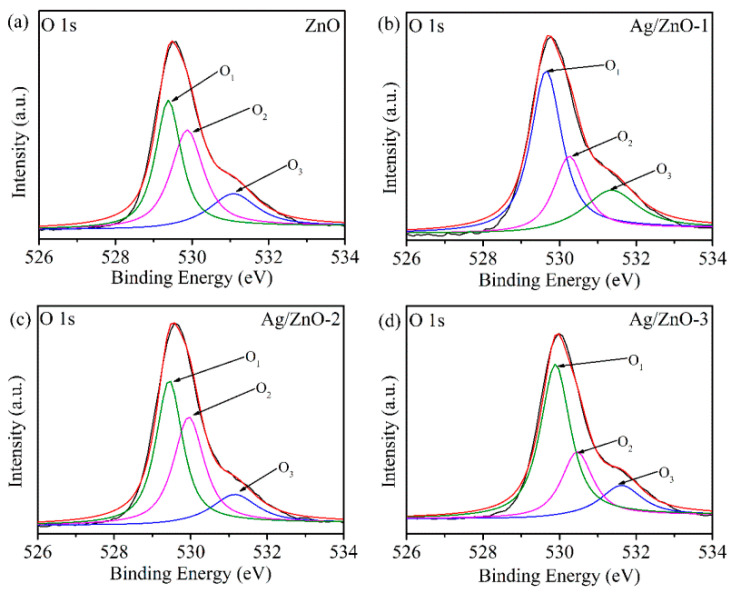
XPS spectra of O 1s of (**a**) ZnO, (**b**) Ag/ZnO-1, (**c**) Ag/ZnO-2 and (**d**) Ag/ZnO-3.

In order to further determine the state of oxygen on the material surface, the O 1s peak XPS spectra of ZnO and Ag/ZnO are fitted in Figure 5. The O 1s of all samples are fitted to three peaks, namely O_1_, O_2_ and O_3_. The O_1_ peak at 529.3 eV is the O^2−^ bonded to Zn^2+^, the O_2_ peak at 530.2 eV is the defect oxygen on the zinc oxide surface, and the O_3_ peak at 531.2 eV is some adsorbed oxygen on the zinc oxide surface. After calculation, it was found that the defect oxygen area ratios of the four samples are 27.8%, 24.1%, 38.9% and 25.6%, respectively. It can be seen that the number of oxygen vacancies on the Ag/ZnO-2 surface is the largest. It is well known that oxygen vacancies can accelerate the decomposition of water molecules into conductive ions [23,24]. For Ag/ZnO-2, recombination of Ag leads to the most oxygen vacancies to dissociate water molecules adsorbed on the surface of zinc oxide to form H_3_O^+^ conductive ions, hence improving humidity sensitivity.

**Figure 6:**
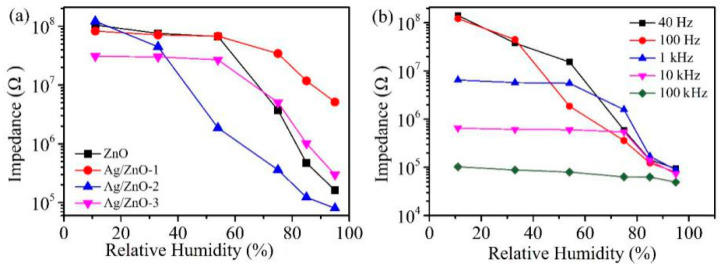
(**a**) Resistance changes of ZnO, Ag/ZnO-1, Ag/ZnO-2 and Ag/ZnO-3 at 11% RH-95% RH; (**b**) Ag/ZnO-2 humidity sensor responses at 40 Hz, 100 Hz, 1 kHz, 10 kHz and 100 kHz.

Figure 6a shows the response curves of ZnO, Ag/ZnO-1, Ag/ZnO-2 and Ag/ZnO-3. We found that the performance of humidity sensor became worse with the increase of Ag^+^ concentration. The humidity sensor shows a good response and the resistance changes more than three orders of magnitude when the molar ratio of Ag^+^: Zn^2+^ is 0.01 (Ag/ZnO-2). It is reported that $\frac{R_{11\%}-R_{95\%}}{R_{95\%}}\times 100\%$ is defined as the response of the sensor [14,25], so it can be calculated that the responses of ZnO, Ag/ZnO-1, Ag/ZnO-2 and Ag/ZnO-3 are 64,300%, 1510%, 151,700% and 10,200%, respectively. Compared with the rGO/ZnO nanorods/Cu humidity sensor (97.79%) reported by Kuntal et al. and the Er:ZnO humidity sensor (impedance change of about three orders of magnitude) reported by Zhang et al., the Ag/ZnO-2 humidity sensor showed a better response [26,27]. The response of Ag/ZnO-2 is probably due to the large amount of oxygen vacancy on surface of ZnO when molar ratio of Ag^+^: Zn^2+^ is 0.01. As an active site, oxygen vacancy can accelerate the decomposition of water molecules adsorbed on surface of ZnO, which makes more water molecules decompose into conductive ions, thus improving the response of Ag/ZnO-2 humidity sensor. In addition, the uniformly dispersed sheet structure of Ag/ZnO-2 can also be used for more water molecules to be adsorbed, which increases the amount of water absorbed on surface of the material and can also enhance the performance of the humidity sensor. Compared with Ag/ZnO-2, ZnO, Ag/ZnO-1 and Ag/ZnO-3 have poorer response. On one hand, there are not enough oxygen vacancies on the surface, which limits the decomposition of water molecules. On the other hand, ZnO, Ag/ZnO-1 and Ag/ZnO-3 particles are agglomerated, resulting in relatively few adsorbed water molecules, which in turn affects humidity sensor response.

In order to determine the optimal test frequency of the Ag/ZnO-3 humidity sensor, we tested the response of Ag/ZnO-1 at 40 Hz, 100 Hz, 1 kHz, 10 kHz and 100 kHz under different relative humidity, and the test results are shown in Figure 6b. Although the response of Ag/ZnO-2 humidity sensor is high at 40 Hz, the overall linearity of the sensor is poor, so 40 Hz cannot be selected as the best test frequency. At 1 kHz, 10 kHz and 100 kHz, the response and linearity of the sensor are poor because the water molecules cannot be polarized at high frequencies, and the polarization of water molecules adsorbed by the sensor cannot keep up with the direction of electric field change in the high-frequency region. Only when the test frequency is 100 Hz, Ag/ZnO-3 humidity sensor shows high response and good linearity. Therefore, we choose 100 Hz as the best test frequency, and all subsequent tests are conducted at this frequency.

**Figure 7:**
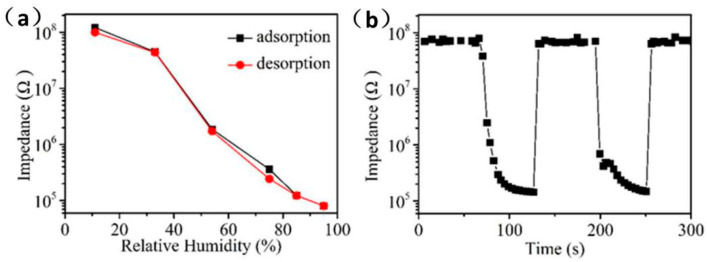
Ag/ZnO-2 humidity sensor (**a**) hysteresis, (**b**) response/recovery time and repeatability test.

The hysteresis, response/recovery time, and repeatability of the humidity sensor are also the main factors that determine the performance of the sensor. Figure 7a shows the hysteresis test of Ag/ZnO-2 humidity sensor in the range of 11% RH to 95% RH. When the environmental RH changes from 11% to 95% RH, the process of the sensor continuously adsorbing water molecules is an adsorption process. Conversely, when the RH is changed from 95% to 11% RH, the process by which the sensor continuously separates from water molecules is a desorption process. It can be observed that the resistance value of adsorption process is almost higher than that of desorption process in whole detection range, which is mainly caused by the endothermic desorption process of water molecules on Ag/ZnO-2 surface is slower than the exothermic adsorption process. The hysteresis error can be calculated according to γH = ±ΔH_max_/2F_FS_, where ΔH_max_ is the maximum hysteresis value and F_FS_ is the full range output of the sensor [28]. It can be calculated that the maximum hysteresis error of the Ag/ZnO humidity sensor is 3%. Compared with previously reported ZnO humidity sensor, the Ag/ZnO-2 humidity sensor also has a low hysteresis error. Ag/ZnO-2 humidity sensor response/recovery time and repeatability test is shown in Figure 7b. It is well known that the time required for the sensor response or recovery process resistance to reach 90% is defined as response or recovery [29]. Response time and recovery time of Ag/ZnO-2 humidity sensor are 36 s and 6 s, respectively. The faster response and recovery speed of Ag/ZnO-2 is mainly due to the abundant oxygen vacancies on its surface, which causes a large number of water molecules to be decomposed quickly. In addition, the distribution of Ag/ZnO-2 is uniform, and the absence of agglomeration will also cause water molecules to quickly detach from the surface of the material. We have continuously tested the adsorption and analysis process of Ag/ZnO-2 humidity sensor in two cycles, and we can see that adsorption and desorption process of the sensor in the two cycles are almost the same, which shows that Ag/ZnO-2 humidity sensor has good repeatability. All the above properties show that Ag/ZnO-2 humidity sensor has the potential to develop a high-performance humidity sensor, and also provides a new idea for the preparation of a new humidity sensor.

**Figure 8:**
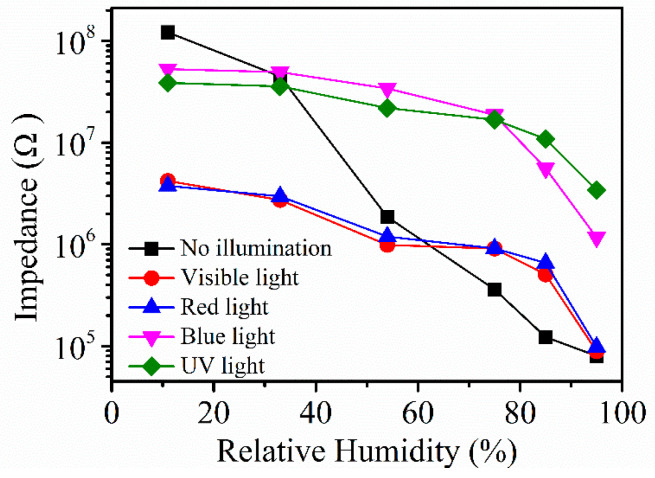
Ag/ZnO-2 resistance response curve under different light.

Figure 8 shows the resistance response curve under different lights. This section studies the effects of light with different wavelength on the resistance of Ag/ZnO-2 and judges the sensing characteristics of the humidity sensor from the situation of the resistance response curve, taking into account that the use of different illuminations can make the surface of the sample obtain a certain amount of energy, and that a small amount of photoelectrons may be generated on the surface of the sample, thereby increasing the conductivity of ZnO. In the experiments, visible (5 W), red (5 W, 546 nm), blue (5 W, 465 nm), and ultraviolet light (5 W, 365 nm) were used to illuminate Ag/ZnO-2 sensors at different relative humidity. We found that in the absence of light, the sensors showed the best response and the best linearity, reflecting that the light affects the performance of the Ag/ZnO-2 humidity sensor, and in the absence of lighting, the synthesis cost of the sensor can undoubtedly be reduced.

**Figure 9:**
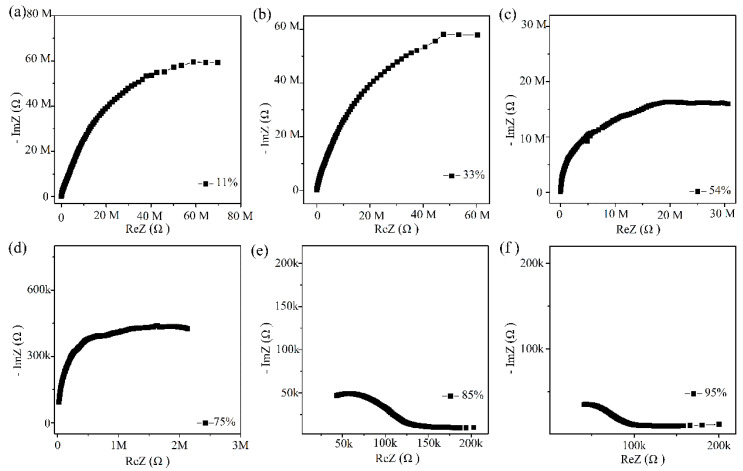
(**a**–**f**) Complex impedance spectroscopy of Ag/ZnO-2 humidity sensor from 11% RH to 95% RH (k refers to 10^3^ and M refers to 10^6^).

The humidity sensing mechanism of Ag/ZnO-2 was studied by complex impedance spectra, as show in Figure 9. At low humidity (11%, 33%, 54% RH), complex impedance spectrum approaches a semicircular shape. In this process, a small amount of water molecules is adsorbed on Ag/ZnO surface in forms of physical and chemical adsorption. The rich oxygen vacancies on the Ag/ZnO-2 surface can accelerate the dissociation of adsorbed water molecules into OH^−^ and H^+^. At this time, a small amount of H_3_O^+^ will be formed, and the protons will conduct jump on the Ag/ZnO surface. When the humidity reaches 75%, 85% and 95% RH, complex impedance spectrum becomes smaller in the semicircle in low frequency region and a straight line gradually appears at the tail. At this time, when a large amount of water molecules is adsorbed on Ag/ZnO-2 surface in the form of chemisorption, a continuous water film is formed. With the increasing number of water molecules and the formation of an ion transport mechanism, H_3_O^+^ continues to migrate onto the surface of the material, enhancing conductivity of the material, thereby improving the performance of the sensor.

## 4. Conclusions

An Ag modified ZnO humidity sensor was successfully prepared, and effects of different Ag concentration on performance of the humidity sensor was studied. Experimental results show that when the molar ratio of Ag^+^: Zn^2+^ in the sample is 1:100, the uniformly distributed Ag particles on ZnO make ZnO have abundant active sites on the surface and more oxygen vacancies, which can capture more water molecules and accelerate the decomposition to form conductive ions, thereby increasing the humidity of Ag/ZnO-2 sensor performance. In the range of relative humidity from 11% to 95%, Ag/ZnO humidity sensor shows better response (151,800%), smaller lag error (3%), faster response and recovery time (36/6 s) and better repeatability. This research has laid the foundation for the development of high-performance metal semiconductor humidity sensors, which are expected to be used for efficient measurement of moisture in the environment.

## Data Availability

Data available in a publicly accessible repository.
